# Apelin-13/APJ system attenuates early brain injury via suppression of endoplasmic reticulum stress-associated TXNIP/NLRP3 inflammasome activation and oxidative stress in a AMPK-dependent manner after subarachnoid hemorrhage in rats

**DOI:** 10.1186/s12974-019-1620-3

**Published:** 2019-12-02

**Authors:** Weilin Xu, Tao Li, Liansheng Gao, Jingwei Zheng, Jun Yan, Jianmin Zhang, Anwen Shao

**Affiliations:** 1 Department of Neurosurgery, Second Affiliated Hospital, School of Medicine, Zhejiang University, 88 Jiefang Rd, Hangzhou, 310009 Zhejiang China; 2 Department of Neurosurgery, Affiliated Tumor Hospital of Guangxi Medical University, Nanning, Guangxi Zhuang Autonomous Region China; 3 Brain Research Institute, Zhejiang University, Hangzhou, Zhejiang China; 4 Collaborative Innovation Center for Brain Science, Zhejiang University, Hangzhou, Zhejiang China

**Keywords:** Early brain injury, SAH, Neuroinflammation, Oxidative stress, Apelin-13, APJ, NLRP3

## Abstract

**Background:**

Neuroinflammation and oxidative stress play important roles in early brain injury following subarachnoid hemorrhage (SAH). This study is the first to show that activation of apelin receptor (APJ) by apelin-13 could reduce endoplasmic reticulum (ER)-stress-associated inflammation and oxidative stress after SAH.

**Methods:**

Apelin-13, apelin siRNA, APJ siRNA, and adenosine monophosphate-activated protein kinase (AMPK) inhibitor-dorsomorphin were used to investigate if the activation of APJ could provide neuroprotective effects after SAH. Brain water content, neurological functions, blood-brain barrier (BBB) integrity, and inflammatory molecules were evaluated at 24 h after SAH. Western blotting and immunofluorescence staining were applied to assess the expression of target proteins.

**Results:**

The results showed that endogenous apelin, APJ, and p-AMPK levels were significantly increased and peaked in the brain 24 h after SAH. In addition, administration of exogenous apelin-13 significantly alleviated neurological functions, attenuated brain edema, preserved BBB integrity, and also improved long-term spatial learning and memory abilities after SAH. The underlying mechanism of the neuroprotective effects of apelin-13 is that it suppresses microglia activation, prevents ER stress from overactivation, and reduces the levels of thioredoxin-interacting protein (TXNIP), NOD-like receptor pyrin domain-containing 3 protein (NLRP3), Bip, cleaved caspase-1, IL-1β, TNFα, myeloperoxidase (MPO), and reactive oxygen species (ROS). Furthermore, the use of APJ siRNA and dorsomorphin abolished the neuroprotective effects of apelin-13 on neuroinflammation and oxidative stress.

**Conclusions:**

Exogenous apelin-13 binding to APJ attenuates early brain injury by reducing ER stress-mediated oxidative stress and neuroinflammation, which is at least partly mediated by the AMPK/TXNIP/NLRP3 signaling pathway.

## Background

Early brain injury (EBI), which is induced within 3 days after subarachnoid hemorrhage (SAH), was reported to be the main cause of poor prognosis of patients with SAH [8, 31]. The underlying mechanisms include neuronal apoptosis, neuroinflammation, oxidative stress, and blood-brain barrier disruption [6, 46]. Increasing studies support the idea that ER stress plays a major role in the post-SAH pathophysiological process [15, 44, 48]. The over activation of ER stress induces calcium release and oxidative stress, which further trigger downstream cascading reactions resulting in inflammation and cellular apoptosis [53]. Li et al. showed that the activation of the thioredoxin-interacting protein (TXNIP)/NOD-like receptor pyrin domain-containing 3 protein (NLRP3) inflammasome could link ER stress to inflammation and cellular apoptosis in brain tissues [16, 17, 19].

Apelin, a peptide with 77 amino acids, is an endogenous ligand for the G protein-coupled receptor APJ [24]. It can be degraded into four shorter active forms, including apelin-12, apelin-13, apelin-17, and apelin-36 ([14] [30];). Among those short forms, apelin-13 displays the strongest biological activity [32, 40]. Recently, apelin-13 has received significant attention from neurologists owing to its neuroprotective effects in the central nervous system. Apelin-13 has displayed wide bioactivity via its receptor APJ, including anti-oxidative stress, anti-apoptosis, and anti-inflammation [26, 40]. However, the role of APJ has not been explored in the SAH rat model.

In addition, Wu et al. showed that apelin-13 could attenuate ER stress-mediated neuronal apoptosis in ischemic stroke [36]. Apelin-13 exerts its neuroprotective effects via its receptor APJ, which further activates adenosine monophosphate-activated protein kinase (AMPK) or suppresses the activation of NLRP3 [12, 43]. In addition, the activation of AMPK leads to phosphorylation and degradation of TXNIP, which could further suppress the overactivation of ER stress and reduce the level of NLRP3 [16, 17, 19]. However, the mechanisms underlying how apelin-13/APJ regulates ER stress and suppresses the activation of NLRP3 after SAH are still unclear.

In this study, we wanted to verify the following hypotheses: (1) SAH could result in the increase of apelin-13, APJ, and p-AMPK levels; (2) apelin-13 could reduce brain edema, BBB disruption, and neurofunctional deficits; (3) knockdown of APJ with siRNA could aggravate neuroinflammation; and (4) selective inhibition of p-AMPK with dorsomorphin could reverse the anti-neuroinflammatory effects of apelin-13.

## Methods

### Animals

All animal experiments were performed according to the Institutional Animal Care and Use Committee of Zhejiang University. The procedures were conducted according to the National Institutes of Health’s Guide for the Care and the Use of Laboratory Animals and the ARRIVE (Animal Research: Reporting In Vivo Experiments) guidelines. We used Sprague-Dawley (SD, male) rats (280–330 g) (SLAC Laboratory Animal Co., Ltd. Shanghai, China) in this study. We kept the rats in a 12-h day/night cycle (22 ± 1 °C; 60 ± 5% humidity). The rats had free access to water and food.

### Experimental design

**Fig. 1:**
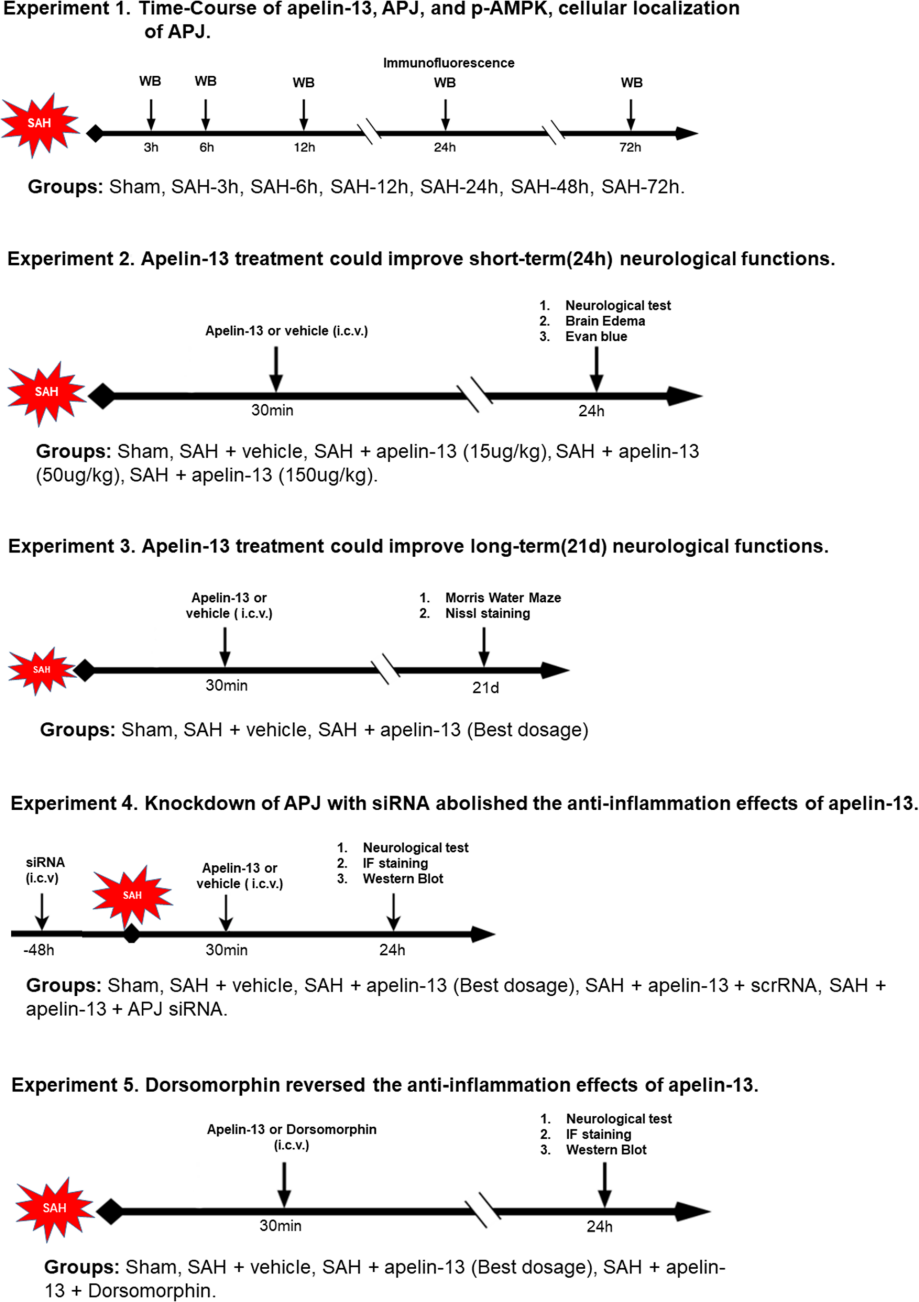
Experimental design and animal groups

This study included five separate experiments using a rat model of SAH (Fig. 1). A total of 312 rats were used in this study, including shared, dead, and excluded animals among each group.

*In the first step*, the expression of endogenous apelin-13, its receptor APJ, and p-AMPK protein levels were assessed in the sham group and at different time points in the SAH groups. Rats were randomly distributed into seven groups: sham, SAH 3 h, SAH 6 h, SAH 12 h, SAH 24 h, SAH 48 h, and SAH 72 h. The ipsilateral/left cerebral cortex from each group was collected for Western blot analysis. The cellular localization of APJ was detected using double immunofluorescence staining (sham and 24 h SAH groups).

*In the second step*, to study the neuroprotective effects of apelin-13, rats were randomly distributed into sham, SAH + vehicle (10 μl sterile saline), SAH + apelin-13 (15 μg/kg), SAH + apelin-13 (50 μg/kg), and SAH + apelin-13 (150 μg/kg). Apelin-13 was intracerebroventricularly given 30 min after SAH. The rats in the sham group received the same procedures as the SAH + vehicle group except injection. Neurological score, brain water content, and Evans blue leakage were measured 24 h after SAH in all groups.

*In the third step*, to explore the effects of treatment with apelin-13 (50 μg/kg, Santa Cruz, sc-351718) on long-term spatial learning and memory, rats were separated into three groups: sham, SAH + vehicle, and SAH + apelin-13. Morris water maze was conducted on days 21–25 after SAH.

*In the fourth step*, we adopted APJ siRNA to further evaluate the role of APJ on the anti-neuroinflammatory effects of exogenous apelin-13. Rats were randomly distributed into sham, SAH + vehicle, SAH + apelin-13 (50 μg/kg), SAH + apelin-13 + scramble siRNA (500 pmol in 10 μl in sterile saline), and SAH + apelin-13 + APJ siRNA (500 pmol in 10 μl in sterile saline). APJ siRNA was injected intracerebroventricularly at 48 h before the induction of SAH. The rats in the sham group received the same procedures as SAH + vehicle group except for injection. The ipsilateral/left cerebral cortex from each group was sampled for Western blot analysis, ROS, and immunofluorescence staining 24 h after SAH.

*In the fifth step*, to assess the role of AMPK on the anti-neuroinflammatory effects of exogenous apelin-13, the selective AMPK inhibitor dorsomorphin (0.1 μmol, in 10 μl 20% DMSO) was administrated (i.c.v.) 30 min after the induction of SAH [41]. Rats were randomly distributed into sham, SAH + vehicle, SAH + apelin-13 (50 μg/kg), and SAH + apelin-13 + dorsomorphin. The ipsilateral/left cerebral cortex from each group was sampled for Western blot analysis, ROS, and immunofluorescence staining 24 h after SAH.

### SAH model in rats

**Fig. 2:**
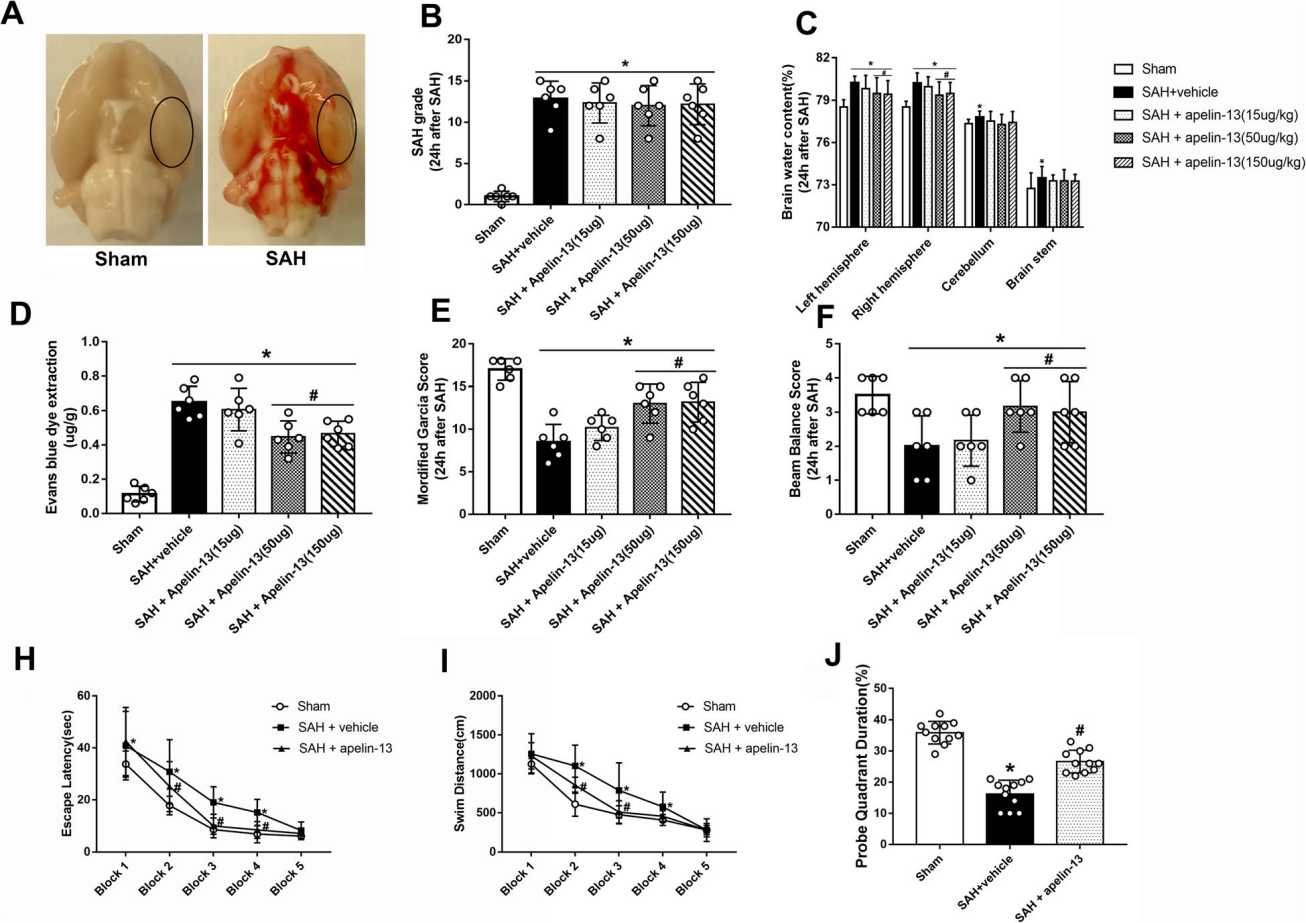
Effects of exogenous apelin-13 on neurological functions, brain edema, and BBB leakage. **a** Representative pictures of brains in Sham and SAH groups. **b** SAH grade of each group. **c** The quantification of brain water content 24 h after SAH. **d** The quantification of Evans blue dye extravasation 24 h after SAH (*n* = 6 for each group). **e**, **f** Modified Garcia score and beam balance score; the effects of apelin-13 on long-term neurobehavioral outcomes after SAH. **h** Escape latency and **i** swim distance of Morris water maze on days 21 to 25 after SAH. **j** Probe quadrant duration. The bars represent the mean ± SD. *P < 0.05 versus sham, ^#^*P* < 0.05 versus SAH + vehicle at 24 h, *n* = 10 per group

The endovascular perforation model was utilized in this study [33]. General anesthesia was induced by peritoneal injection with pentobarbital (40 mg/kg) before the carotid artery (CA) and its bifurcation was necessarily exposed, and then a 4-0 sharpened nylon suture was inserted in the external carotid artery (ECA), which was dissected in advance. The suture marched along the internal carotid artery (ICA) to arrive at the intracranial bifurcation of the anterior cerebral artery (ACA) and middle cerebral artery (MCA), where a perforation was executed (Fig. 2a). All the perforations were performed on the left side. Therefore, the ventral side of the left hemisphere was collected for further study. Additionally, the degree of SAH was quantitatively assessed via a new grading system ([34], Additional file 1: Figure S1). The rats in the sham group received the same procedures except perforation. The rat with SAH grade less than 7 was excluded from this study.

### Mortality and behavior assessment

The timing of tests was set at 24 h after SAH induction, depending on the time-course in which the content of apelin and APJ peaked at the point-in-time. Therefore, we calculated the mortality rate and assessed the neurological functions at 24 h using the previously described modified Garcia scoring system and beam balance test [11]. The total Garcia score was graded with a scale ranging from 1 to 18. Long-term neurobehavior assessments were conducted with Morris water maze on 21–25th day after SAH. We performed the water maze test according to a previous report [10, 21, 47].

### Brain water content

We used the wet-dry method to evaluate the brain water content 24 h after SAH. Briefly, the rats received euthanasia and the brains were collected quickly and then separated into four parts: left hemisphere, right hemisphere, cerebellum, and brain stem. Afterwards, each part of the brain was weighed (wet weight). Next, each part was put in an oven for 72 h (105 °C, dry weight). We then calculated the brain water content as follows: [(wet weight − dry weight)/(wet weight)] × 100% [49, 50].

### Evans blue staining

Evans blue (EB) staining was applied to evaluate the blood-brain barrier integrity. Two percent EB solution (8 ml/kg, Sigma-Aldrich) was intraperitoneally injected after anesthesia. After 24 h, the rats received trans-cardiac perfusion with 0.1 M PBS. Next, the brain was removed and homogenized in 50% trichloroacetic acid. The sample was incubated in a water bath (50 °C) for 48 h and centrifuged at 15,000×*g* for 30 min. The supernatant was detected by spectrofluorophotometry at 620 nm [51].

### Immunohistochemistry staining

The rats received trans-cardiac perfusion with 0.1 M PBS after anesthetization, followed by 4% paraformaldehyde (pH = 7.4). We then collected the brains and put them into 4% PFA for post-fixation (4 °C, 24 h). Then, the brains were immersed in sucrose solution (30%, 2 days). Next, the brains were coronally sliced into 10 μm sections, which were then fixed on slides and used for immunofluorescence staining, and blocked with 5% normal donkey serum at room temperature for 2 h and then incubated with primary antibodies at 4 °C overnight: APJ (1:100, Santa Cruz sc-517300), IL-1β (1:100, Santa Cruz sc-52012), Iba-1 (1:500, Abcam ab5076), GFAP (1:500, Abcam ab7260), and NeuN (1:500, Abcam ab177487). Secondary antibodies were then applied at room temperature for 2 h. Finally, the sections were assessed with a fluorescence microscope (Olympus, Tokyo, Japan) and images were further processed using Photoshop 13.0 (Adobe Systems Inc., Seattle, WA, USA). The number of Iba-1 and myeloperoxidase (MPO) positive cells was counted in three different fields in the ipsilateral cortex from five random coronal sections per brain using a magnification of × 200 over a microscopic field of 0.01 mm^2^, and data were expressed as cells/field.

### Small interfering RNA and intracerebroventricular injection

Intracerebroventricular injection was performed according to a previous report [47]. After the rats were anesthetized, we used a cranial drill to make a burr hole at 1 mm posterior to the bregma and 1.5 mm right lateral to the midline. A total volume of 10 μl (500 pmol, sterile saline) of rat APJ siRNA (Thermo Fisher Scientific, USA) was then injected into the right ventricle (3.5 mm depth below the skull) with a pump at the rate of 0.5 μl/min 48 h before SAH. Moreover, the same volume of scramble siRNA (Thermo Fisher Scientific, USA) was intracerebroventricularly injected as a negative control. The needle was kept in place for 5 min. Finally, the burr hole was closed with bone wax and the incision was sealed with sutures. We chose this timepoint based on several papers previously reported [25, 45]. The siRNA needs at least 24 h to exert its knockdown functions. Therefore, the 48th hour before SAH is a reasonable timepoint for giving siRNA.

### Western blot analysis

The rats first received transcardiac perfusion with 0.1 M PBS after being anesthetized. The ipsilateral/left cerebral cortex of the rat was then sampled and processed as previously being reported [23]. Briefly, we collected the left basal cortical specimen 24 h after SAH and further processed the samples. Protein (40 μg) from each sample was added for electrophoresis (80 V, 30 min; 120 V, 50 min) and then transferred to the polyvinylidene fluoride membranes at 250 mA for 1 h. Then, the membrane was blocked for 1 h at room temperature with 5% non-fat blocking grade milk (Bio-Rad, Hercules, CA, USA). Next, the membrane was incubated with primary antibodies overnight (4 °C): apelin-13 (1:500, Phoenix Pharmaceuticals H-057-30), APJ (1:500, Santa Cruz sc-517300), Bip/GRP78 (1:2000, Abcam ab21685), TXNIP (1:2000, Abcam ab188865), AMPK (1:1000, Cell signaling #5832), p-AMPK (1:1000, Cell signaling #2535), IL-1β (1:2000, Santa Cruz SC-23459), TNF-α (1:5000, Abcam ab6671), NLRP3 (1:1000, NOVUS, CO), and β-actin (1:5000, Abcam ab8226). Secondary antibodies (1:10000, Zhongshan Gold Bridge) were then applied at room temperature for 1 h. Finally, we used ECL Plus chemiluminescence reagent kit (Amersham Bioscience, Arlington Heights, IL) to expose and detect the protein. Then, ImageJ software (NIH) was used to measure intensity. The results were displayed as relative density (grayscale value of the target proteins/β-actin or total proteins).

### Evaluation of ROS level

We detected the level of ROS in brain tissues using a ROS assay kit (JianCheng, China). Briefly, the brain tissues were lysed in 0.01 mol/L PBS with centrifugation at 4500×*g* for 10 min. We then collected the supernatant (190 μl) and mixed it with DCFH-DA (10 μl, 1 mol/L) in a micro-well at room temperature for 30 min. Next, fluorophotometry was used to detect the sample. We measured the protein levels of different samples using a detergent-compatible protein assay kit (Bio-Rad, Hercules, CA, USA). The ROS levels were measured in the form of fluorescence/milligram protein.

### Statistical analysis

Results were displayed as the mean ± SD. SPSS 22.0 software was applied to analyze the data (IBM, USA). First, we assessed the normality of the data. If the data met the requirement of satisfied normality and homogeneity of variance, one-way analysis of variance (ANOVA) is followed by multiple comparisons between different groups using Tukey’s post hoc test. For the data that failed the normality test, non-parametric statistics was applied. Additionally, two-way repeated-measures ANOVA was applied to analyze the data of long-term neurological functions and brain water content. Statistical significance was set at *P* < 0.05.

## Results

### Physiological data

The data regarding physiology such as body temperature, heart rate, blood pressure, blood glucose, PO_2_, and PCO_2_ were monitored during the study, and no significant difference between any two groups was noted (Additional file 1: Table S2).

### Mortality rates and SAH grade score

The total number of rats used in this study was 312; 53 rats were in the sham group, 18 rats were treated as naïve groups, and 241 rats received perforation surgery. The overall mortality of SAH was 16.18%. No rats died in the sham group, and 9 rats were excluded owing to mild SAH (SAH grade < 7) (Additional file 1: Table S1). No significant differences in SAH grade among all SAH groups were observed (Fig. 2b). Blood clots were mainly observed around the circle of Willis and ventral brain stem after SAH induction. We chose the region of basal cortex of the left hemisphere for immunochemistry staining and Western blot analysis (Fig. 2a).

### Brain edema, BBB permeability, and neurological functions at 24 h after SAH

Three dosages of the apelin-13 (15 μg/10 μl, 50 μg/10 μl, 150 μg/10 μl) were intracerebroventricularly administered 1 h after SAH. Brain water content and BBB integrity were evaluated 24 h after SAH. The results showed that brain water content in the SAH + vehicle group was significantly increased compared to the sham group (*P* < 0.05, Fig. 2c). The use of middle and high doses of apelin-13 significantly reduced the brain edema in the left and right hemispheres (*P* < 0.05 versus SAH + vehicle, *n* = 6, Fig. 2c), while no differences between these two groups were observed. For the cerebellum and brain stem, no significant differences were observed after the use of apelin-13 (*P* > 0.05 versus SAH + vehicle, Additional file 1: Figure S2). In addition, the Evans blue dye significantly leaked in the SAH + vehicle group compared to the sham group (*P* < 0.05, Fig. 2d). However, rats treated with middle and high doses of apelin-13 had less dye leakage compared to the rats in the SAH + vehicle group (*P* < 0.05, Fig. 2d). The induction of SAH greatly aggravated the neurological deficits, which were significantly relieved by middle and high doses of apelin-13 (Fig. 2e, f). However, there was also no significant difference between the groups receiving middle and high doses of apelin-13. Therefore, we chose the middle dose of apelin-13 for future experiments.

### Administration of apelin-13 improved long-term spatial learning and memory after SAH

The escape latency and swim distance for the rats to find the platform significantly increased in the SAH + vehicle group compared to the sham group (Fig. 2h, i). After treatment with apelin-13, there was a significant decrease in escape latency on blocks 2, 3, and 4 as well as a shorter swim distance on blocks 2 and 3 compared to the SAH + vehicle group (Fig. 2h, i). Regarding the probe quadrant trial, rats in the SAH + vehicle group stayed a shorter time in the target quadrant compared to rats in the sham group. However, rats in the SAH + apelin-13 group stayed a longer time than the rats in the SAH + vehicle group (Fig. 2j).

### Expression level of apelin-13, APJ, and p-AMPK after SAH

**Fig. 3:**
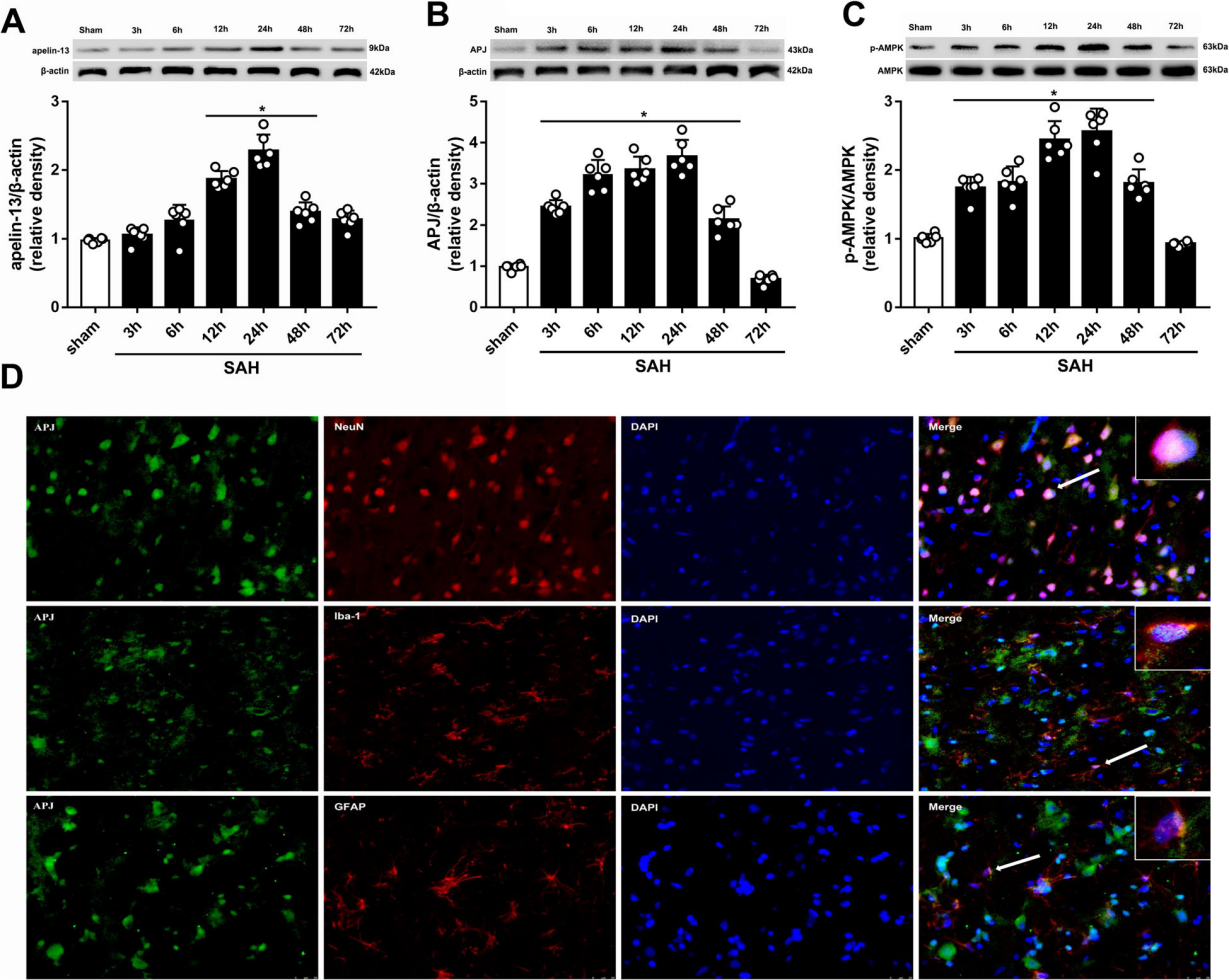
Expression of apelin-13, APJ, and p-AMPK. **a** Representative Western blot images and quantitative analyses of apelin-13 time-course from the left hemisphere after SAH. **b** Representative Western blot images and quantitative analyses of APJ time course from the left hemisphere after SAH. **c** Representative Western blot images and quantitative analyses of p-AMPK time course from the left hemisphere after SAH; *n* = 6 for each group. The bars represent the mean ± SD. *P < 0.05 versus sham. **d** Representative microphotographs of immunofluorescence staining showing localization of APJ (green) with NeuN, iba-1, and GFAP (red) 24 h after SAH (*N* = 2 for each group). Scale bar = 50 μm

The results showed that the level of apelin-13 increased at 12 h and peaked at 24 h after SAH (Fig. 3a), while the levels of APJ and p-AMPK started to increase at 6 h and peaked at 24 h after SAH (Fig. 3b, c).

### Cellular location of APJ after SAH

The results of double immunofluorescence staining suggested that APJ was expressed on all types of cells. The percentage that APJ could be colocalized with the neuron or microglia is much higher than that of astrocytes in the cortex at 24 h after SAH (Fig. 3d).

### Anti-oxidative stress and anti-inflammation effects of exogenous apelin-13 is partly abolished by APJ siRNA at 24 h after SAH

**Fig. 4:**
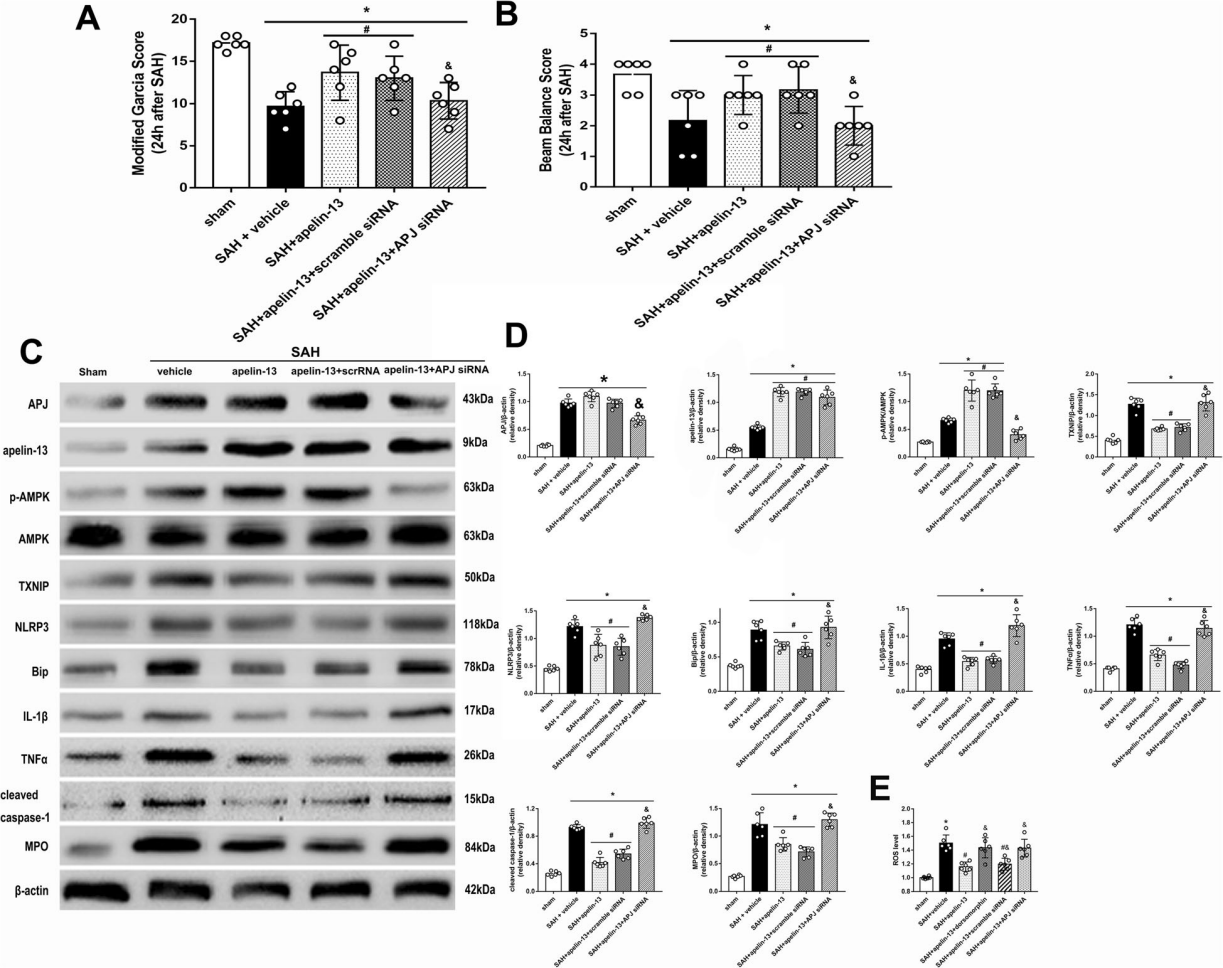
Knockdown of APJ abolished the neuroprotective effects of exogenous apelin-13 24 h after SAH. **a**, **b** Modified Garcia score and beam balance score. **c** Representative Western blot images. **d** Quantitative analyses of APJ, apelin-13, p-AMPK, TXNIP, NLRP3, Bip, IL-1β, TNFα, cleaved caspase-1, and MPO. *n* = 6 for each group. The bars represent the mean ± SD. *P < 0.05 versus sham, ^#^*P* < 0.05 versus SAH + vehicle, ^&^*P* < 0.05 versus SAH + apelin-13. **e** The level of ROS

The administration of exogenous apelin-13 significantly improved the neurological functions after SAH (*P* < 0.05, Fig. 4a, b). In addition, exogenous apelin-13 further increased the level of p-AMPK, which suppressed the expression of downstream targets, including TXNIP, NLRP3, Bip, cleaved caspase-1, IL-1β, and TNFα, and also reduced the MPO levels (*P* < 0.05, Fig. 4c, d) compared to the SAH + vehicle group. However, the knockdown of APJ with APJ siRNA partly offset anti-oxidative stress and anti-inflammation effects of exogenous apelin-13. The results showed that the knockdown of APJ inhibited the expression of APJ and p-AMPK 24 h after SAH and increased the levels of TXNIP, NLRP3, Bip, cleaved caspase-1, IL-1β, TNFα, MPO, and ROS (*P* < 0.05, Fig. 4c–e). Moreover, exogenous apelin-13 decreased the level of ROS, which is also partly abolished by APJ siRNA (*P* < 0.05, Fig. 4e).

### AMPK inhibitor, dorsomorphin, reversed the anti-inflammatory effects of apelin-13 24 h after SAH

**Fig. 5:**
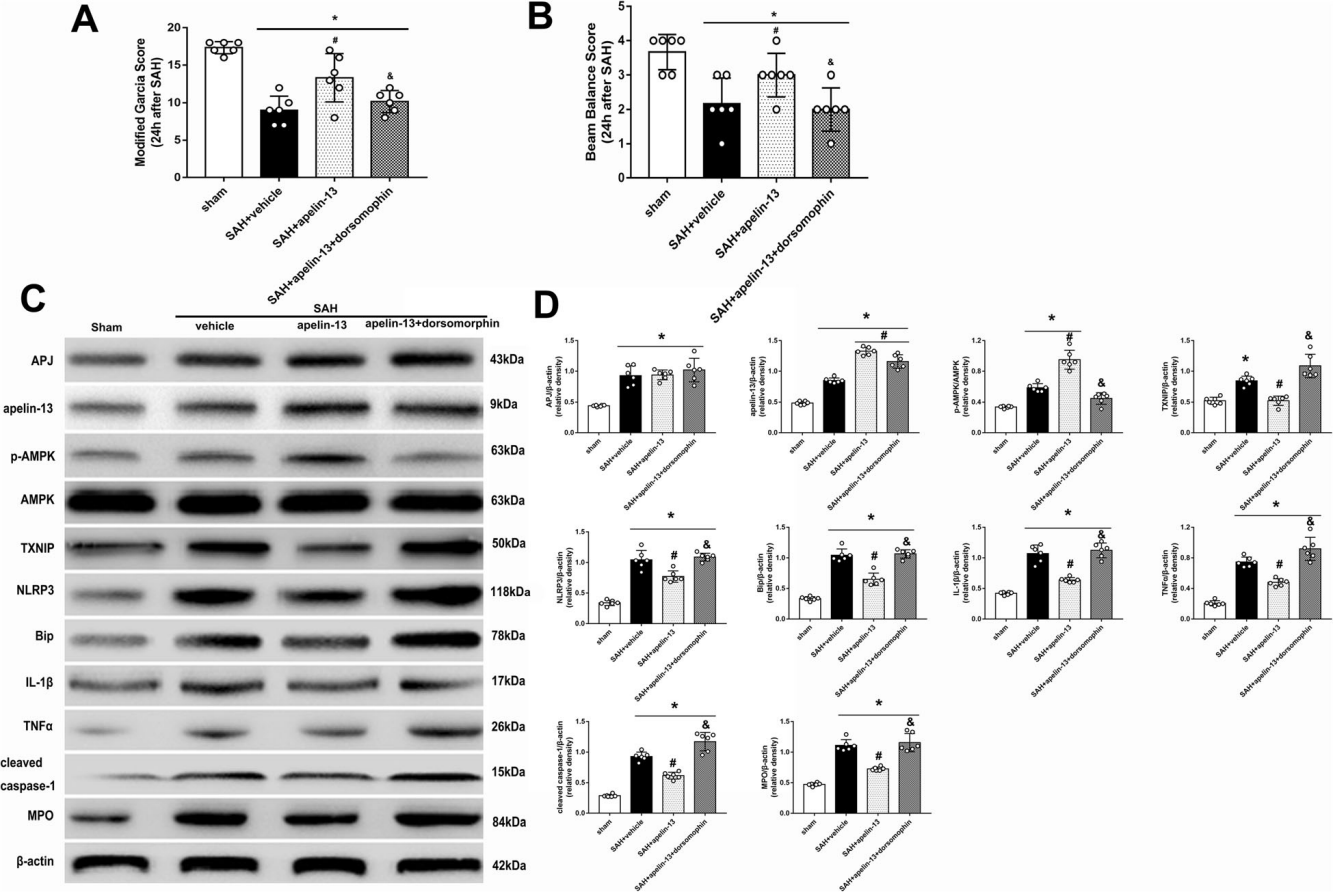
AMPK inhibitor, dorsomorphin, reversed the anti-inflammatory effects of apelin-13 24 h after SAH. **a**, **b** Modified Garcia score and beam balance score. **c** Representative Western blot images. **d** Quantitative analyses of APJ, apelin-13, p-AMPK, TXNIP, NLRP3, Bip, IL-1β, TNFα, cleaved caspase-1, and MPO. *n* = 6 for each group. The bars represent the mean ± SD. *P < 0.05 versus sham, ^#^*P* < 0.05 versus SAH + vehicle, ^&^*P* < 0.05 versus SAH + apelin-13

**Fig. 6:**
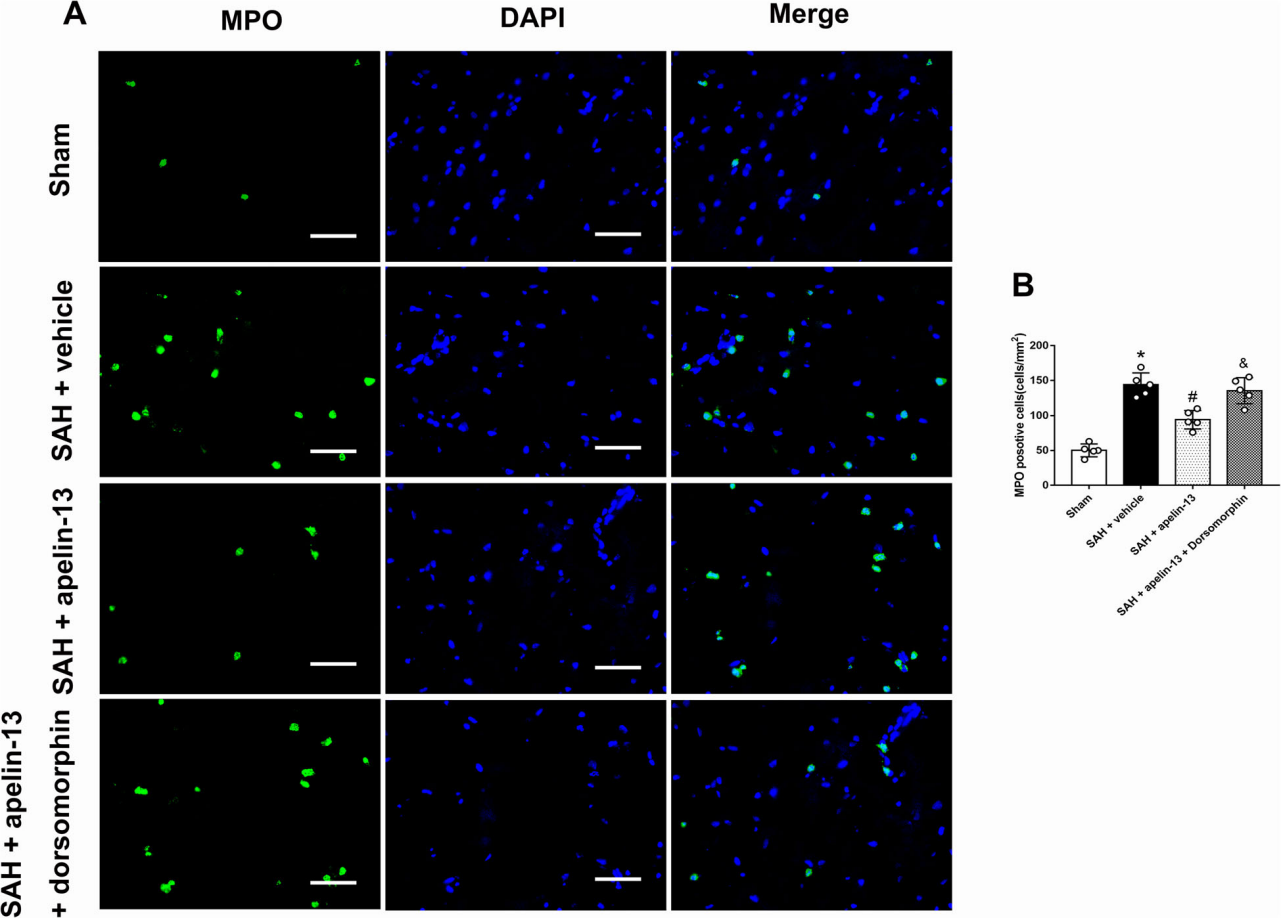
The effects of apelin-13 on neutrophil infiltration after SAH. **a** Representative images of immunofluorescence staining of MPO (green) with DAPI (blue) from the left hemisphere. **b** Quantitative analyses of MPO-positive cells from the left hemisphere 24 h after SAH. *n* = 5 for each group

**Fig. 7:**
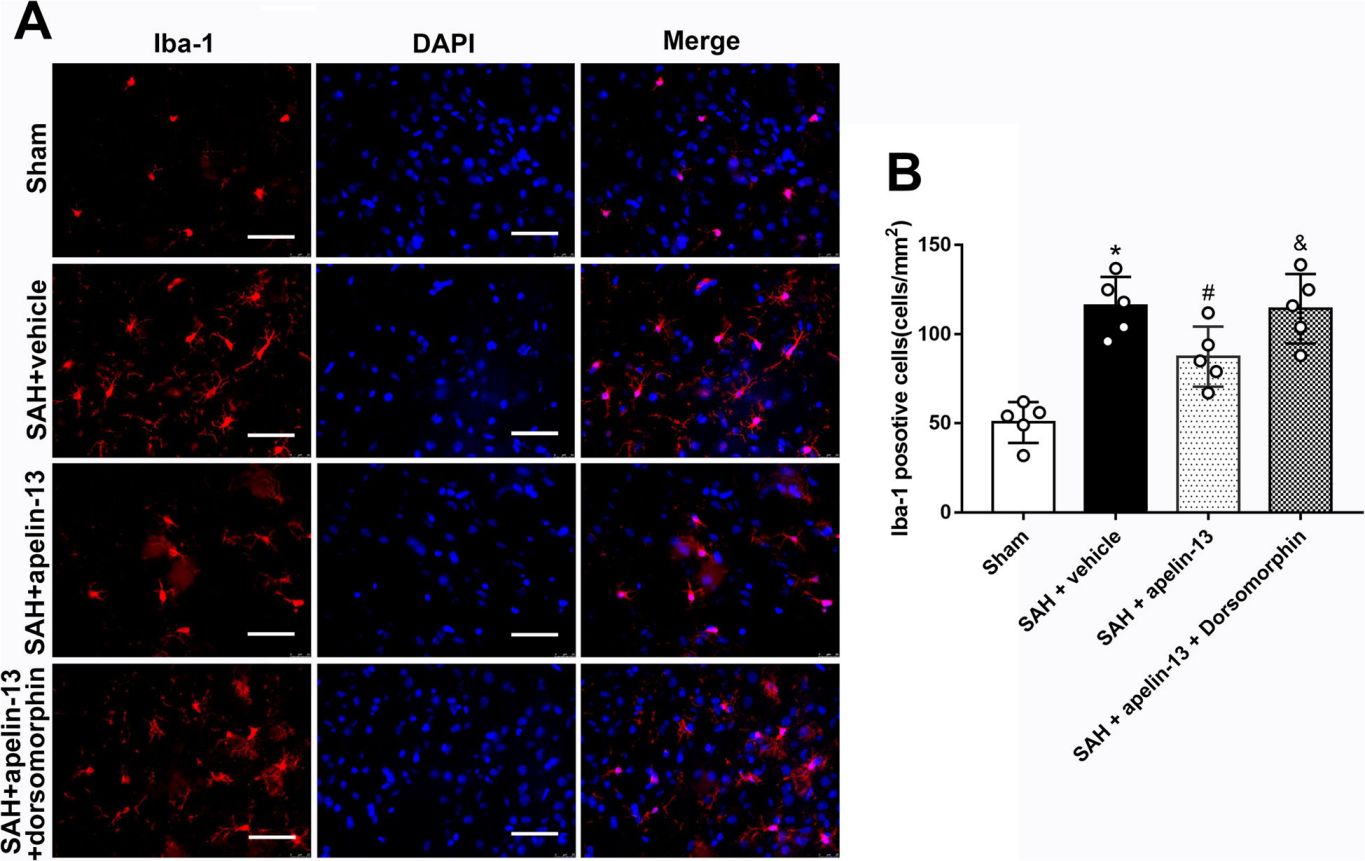
The effects of apelin-13 on microglia/macrophage activation after SAH. **a** Representative images of immunofluorescence staining of Iba-1 (green) with DAPI (blue) from the left hemisphere. **b** Quantitative analyses of Iba-1-positive cells from the left hemisphere 24 h after SAH. *n* = 5 for each group

**Fig. 8:**
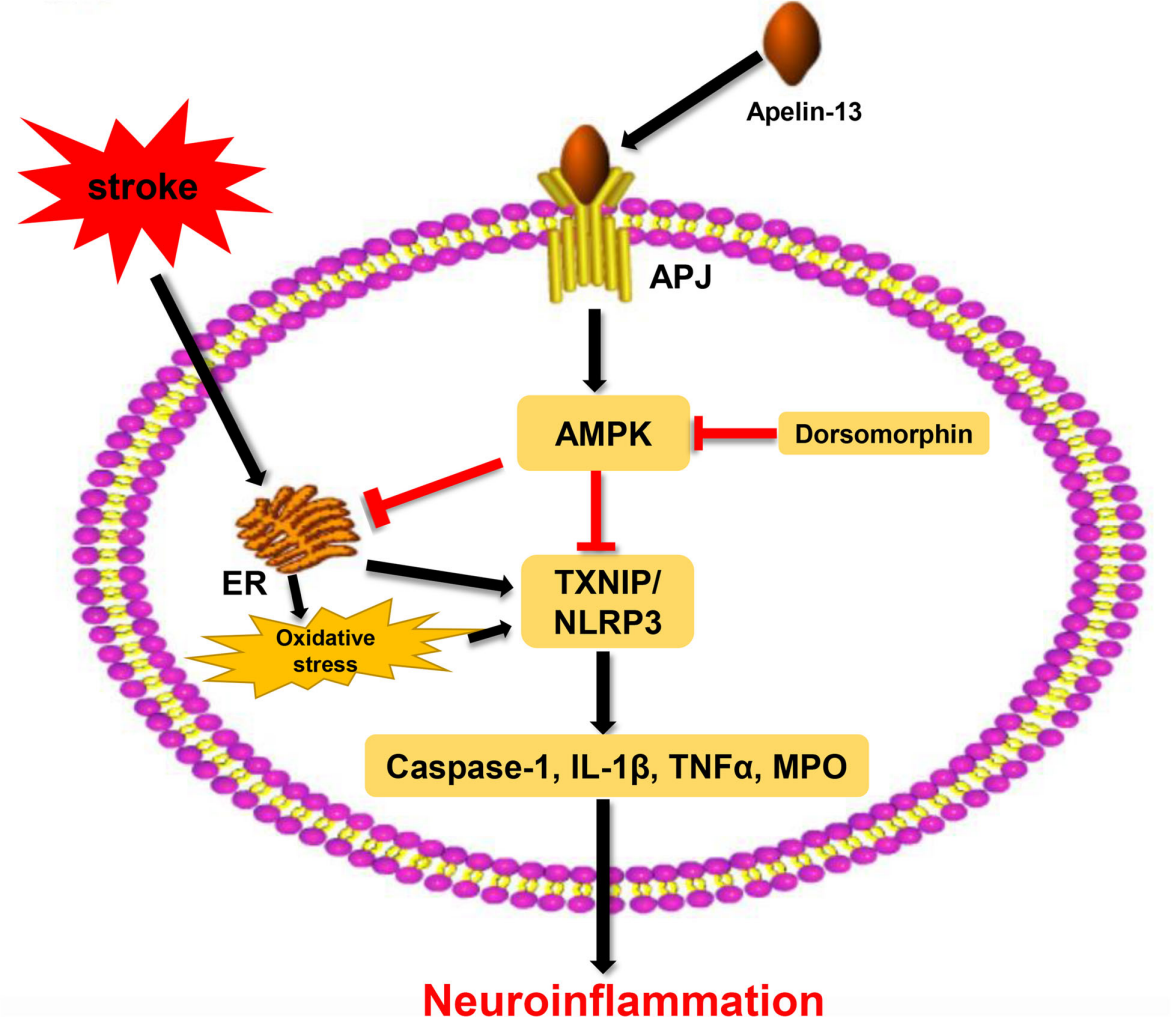
The effects of dorsomorphin on the rats with SAH. **a** Representative Western blot images. **b** Quantitative analyses of p-AMPK, IL-1β, TNFα, cleaved caspase-1, MPO. *n* = 6 for each group. The bars represent the mean ± SD. *P < 0.05 versus sham, ^#^*P* < 0.05 versus SAH + vehicle

The AMPK inhibitor, dorsomorphin, was used to further verify the neuroprotective pathway of apelin-13 after SAH. Dorsomorphin treatment significantly aggravated the neurological deficits (*P* < 0.05, Fig. 5a, b). Furthermore, the administration of dorsomorphin reduced the level of p-AMPK and increased the levels of TXNIP, NLRP3, Bip, cleaved caspase-1, IL-1β, TNFα, MPO, and ROS (*P* < 0.05, Figs. 4e and 5c, d). MPO positive and iba-1 positive cells were also significantly increased after the use of dorsomorphin (*P* < 0.05, Figs. 6a, b and 7a, b). Besides, we did an additional experiment where the dorsomorphin is given alone to see the effects of dorsomorphin. The results showed that the inhibition of AMPK significantly increased the inflammatory molecules (IL-1β, TNFα, MPO, etc.; *P* < 0.05, Fig. 8a, b).

### Assessment of the depletion efficiency of APJ siRNA with naïve rats

In order to test the depletion efficiency of APJ siRNA, we intraventricularly injected APJ siRNA in naïve rats. The results showed that APJ siRNA reduced the level of APJ by 46% on average (Additional file 1: Figure S3).

## Discussion

In this study, we explored the neuroprotective effects of apelin-13 through a new mechanism mediated by the APJ/AMPK/TXNIP/NLRP3 pathway in experimental SAH in rats. The novel findings of this study include the following: (1) endogenous apelin-13, APJ, and p-AMPK levels were significantly increased and peaked in the brain 24 h after SAH; APJ was mainly expressed in the microglia and neurons; (2) administration of exogenous apelin-13 significantly alleviated neurological functions, attenuated brain edema, and preserved BBB integrity; (3) treatment with apelin-13 significantly improved long-term spatial learning and memory after SAH; (4) exogenous apelin-13 suppressed microglia activation, prevented ER stress from overactivation, and reduced the levels of TXNIP, NLRP3, Bip, cleaved caspase-1, IL-1β, TNFα, MPO, and ROS; (5) siRNA knockdown of APJ also abolished the neuroprotective effects of apelin-13 on neuroinflammation and oxidative stress; and (6) the AMPK inhibitor dorsomorphin also reversed the neuroprotective effects of apelin-13 24 h after SAH. Taken together, the results show that exogenous apelin-13 binding to APJ can attenuate early brain injury by reducing ER stress-mediated oxidative stress and neuroinflammation, which is at least partly mediated by the AMPK/TXNIP/NLRP3 signaling pathway.

Increasing studies show the important roles of neuroinflammation and ER stress-mediated oxidative stress in early brain injury after SAH [7, 42]. ER is an organelle that mainly manipulates protein synthesis and further processing. Insults that perturb ER function leads to ER stress [27], which could be induced by inflammation, oxidative stress, or mitochondrial calcium overloading [18]. After induction of SAH, blood immediately spreads through the subarachnoid space and then covers the cerebral cortex with a thick blood clot [22]. The resulting accumulations of cell breakdown components, dysfunctional organelles, inflammatory factors, ROS, and other cytokines can inhibit normal protein folding, which activates UPR/ER stress and finally causes irreversible neurological deficits [42]. In turn, the overactivation of ER stress aggravates the inflammatory response and oxidative stress, including further microglia activation and leukocyte infiltration into the brain, which subsequently trap in a vicious circle to exacerbate brain injury after stroke [13, 39]. Therefore, suppressing microglia activation and neutrophil infiltration and reducing oxidative stress are beneficial to ameliorate early brain injury after SAH.

Apelin-13 has been reported to provide neuroprotection against cerebral ischemic stroke via anti-neuroinflammation, anti-oxidative stress, or preventing ER stress from overactivation [3, 4, 43]. Leeper et al. also found that apelin-13 significantly reduced aneurysm formation by decreasing proinflammatory cytokine and chemokine activation [20]. Zhang et al. reported that apelin-13 could attenuate cisplatin-induced cardiotoxicity by reducing ROS-mediated DNA damage [49, 50]. In the present study, we found that the administration of apelin-13 significantly improved neurological functions and reduced the inflammatory response, neutrophil infiltration, and oxidative stress including the levels of Bip, IL-1β, TNFα, MPO, and ROS. These observations suggest that apelin-13 could suppress neuroinflammation and oxidative stress by inhibiting the expression of inflammatory molecules and preventing overactivation of the ER stress response following SAH.

Apelin-13 exerts its functions through binding to its receptor APJ. Apelin-13 was reported to protect against multiple organ injury following hemorrhagic shock by binding to APJ and decreasing the number of inflammatory cells and swollen mitochondria in cells [29]. In the model of ischemic stroke, the apelin-13/APJ system suppressed the activation of microglia and astrocyte and reduced neutrophil infiltration [40]. In the current study, the results of double immunofluorescence staining showed that APJ was expressed in microglia and neurons. Moreover, the knockdown of APJ with specific siRNA significantly abolished the anti-inflammation and anti-oxidative stress property of exogenous apelin-13, which could suppress microglia activation and diminish pro-inflammatory TNF-α and IL-1β levels as well as the levels of ROS and MPO. Therefore, it is reasonable to speculate that APJ mediates apelin-13-induced anti-inflammation and anti-oxidative effects after SAH.

**Fig. 9:**
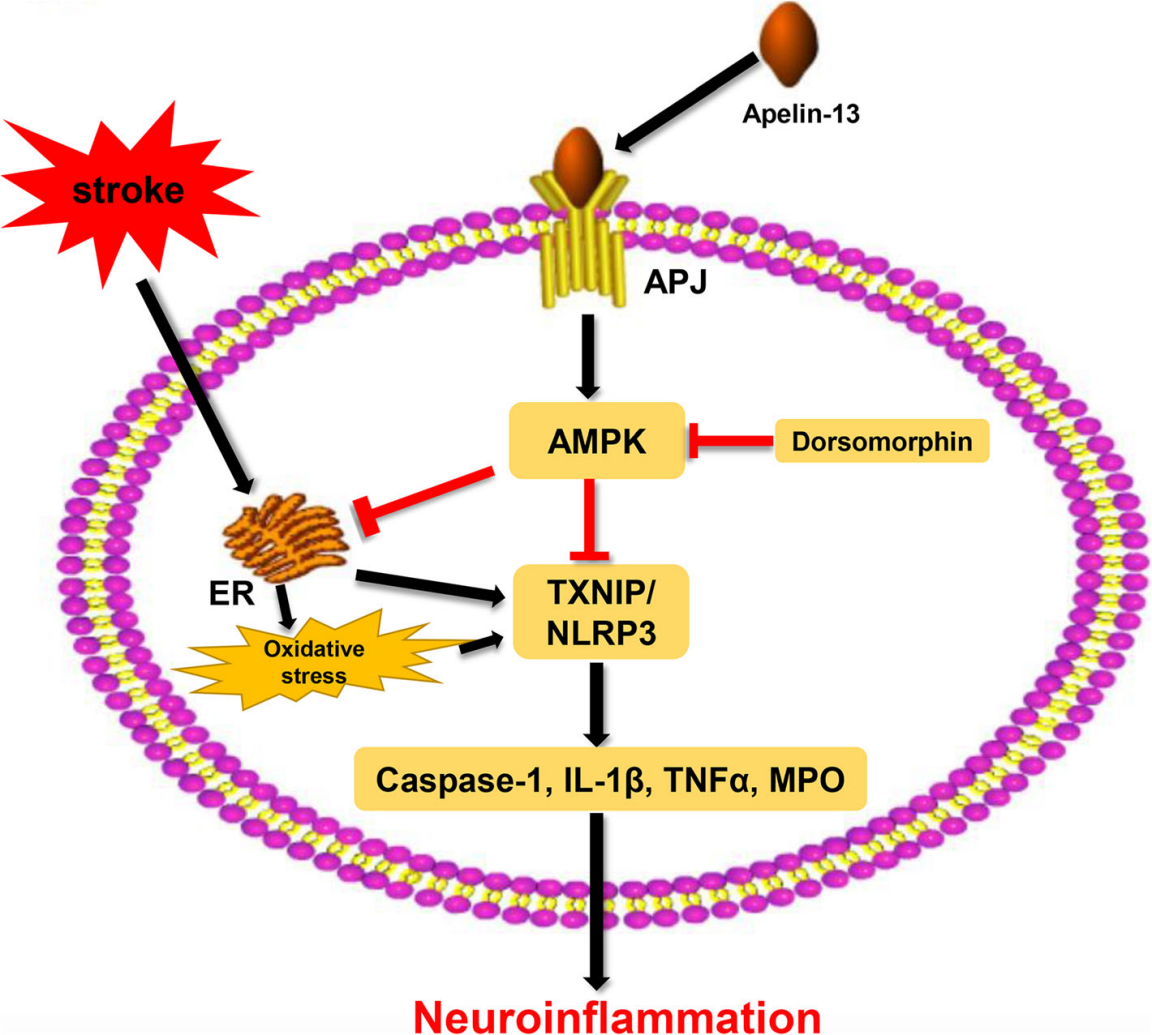
The potential molecular mechanisms of apelin-13/APJ system-mediated anti-inflammatory and anti-oxidative effects via suppression of ER stress-associated AMPK/TXNIP/NLRP3 inflammasome activation

AMPK has been reported to be the key factor in regulating the inflammatory response and ER stress [16, 17, 19, 37]. An et al. reported the activation of AMPK increased neuronal apoptosis and brain injury after SAH [2]; however, more studies hold the opinion that phosphorylation of AMPK provides neuroprotection in acute stroke attack as follows. The level of AMPK was increased after stroke, which could further suppress the activation of microglia and reduce the infiltration of neutrophil into brain tissues [38]. Increased AMPK reinforced Nrf2-mediated antioxidant pathways [1]. Ying et al. found that AMPK could attenuate glutamate neurotoxicity in the hippocampus by suppressing the ER stress-mediated TXNIP/NLRP3 inflammasome [16]. Moreover, enhanced AMPK phosphorylation reduced ROS and ER stress-mediated TXNIP/NLRP3 inflammasome [52]. Recently, increasing evidence has indicated the importance of the NLRP3 inflammasome in the post-SAH inflammatory response [9, 28]. Once activated, the NLRP3 inflammasome causes transformation of procaspase-1 into cleaved caspase-1 and maturation of IL-1β and IL-18, which subsequently contributes to inflammation after SAH [9]. In addition, some studies showed that AMPK is a downstream target of apelin-13-mediated anti-inflammation and anti-oxidative stress during ischemic injury in the brain and heart [35, 43]. In addition, Apelin-13 attenuated the systemic inflammatory response and promoted survival after severe burn by inhibiting the activation of the NLRP3 inflammasome in rats [5]. In this study, we observed that the levels of APJ and AMPK increased after SAH. However, the extent of endogenous APJ and AMPK upregulation was not enough to reduce the level of the NLRP3 inflammasome and inflammatory cytokines. After the use of exogenous apelin-13, the level of AMPK was further increased, which significantly inhibited the activation of the NLRP3 inflammasome and reduced the levels of Bip, cleaved caspase-1, IL-1β, TNF-α, MPO, and ROS. However, the AMPK inhibitor reversed the anti-inflammatory and anti-oxidative stress effects and upregulated the expression of NLRP3, cleaved caspase-1, IL-1β, TNFα, MPO, and ROS, thereby worsening the neurological deficits. Therefore, our findings supported the hypothesis that the anti-inflammatory and anti-oxidative stress effects of exogenous apelin-13 is mediated at least in part through the AMPK/TXNIP/NLRP3 signaling pathway after SAH (Fig. 9).

Although this study verified the value of apelin-13 in a novel mechanism mediated via suppression of the AMPK/TXNIP/NLRP3 signaling pathway, some limitations could not be ignored. First, the apelin-13/APJ system can display its neuroprotective effects following SAH in many ways; however, only the AMPK-dependent pathway was studied. Second, only the anti-inflammatory and anti-oxidative characteristics of the apelin-13/APJ system were evaluated in this study, without further investigation of its roles in apoptosis or autophagy. Therefore, further studies focusing on other roles of the apelin-13/APJ system in SAH and more characteristics of the apelin-13/APJ system need to be performed.

## Conclusions

Our study showed that exogenous apelin-13 binding to APJ improves neurological functions and attenuates early brain injury after SAH by reducing ER stress-mediated oxidative stress and neuroinflammation, which is at least partly mediated by the AMPK/TXNIP/NLRP3 signaling pathway. Therefore, apelin-13/APJ system can be a promising therapeutic target in the treatment of SAH.

## Supplementary information

**Additional file 1: Table S1** Study design and animal usage. **Table S2.** Rats physical data after surgeries. **Figure S1.** The ventral side was divided into six parts. SAH severity score: a grade from 0-3 is dependent on the amount of blood clot in each segment as follows: grade 0:no blood clot; 1: minimal blood clot; 2: moderate blood clot with recognizable arteries; 3: blood clot obliterating all arteries. The SAH grade was the total scores of the six parts, with minimal score of 0 and maximal score of 18. **Figure S2.** Effects of apelin-13 on brain edema: the quantification of brain water content of cerebellum and brain stem at 24 h after SAH. **Figure S3.** Depletion Efficiency of APJ siRNA with Naïve Rats. (A) Representative Western blot images. (B) Quantitative analyses of APJ. n=6 for each group. The bars represent the mean ± SD. *p<0.05 versus naïve.

## Data Availability

The datasets analyzed during the current study are available from the corresponding author on reasonable request.

## Abbreviations

SAH: Subarachnoid hemorrhage
APJ: Apelin receptor
TXNIP: Thioredoxin-interacting protein
NLRP3: NOD-like receptor pyrin domain-containing 3 protein
AMPK: Adenosine monophosphate-activated protein kinase
ER: Endoplasmic reticulum
BBB: Blood-brain barrier
MPO: Myeloperoxidase
ROS: Reactive oxygen species
EBI: Early brain injury
CA: Carotid artery
ECA: External carotid artery
ICA: Internal carotid artery
ACA: Anterior cerebral artery
MCA: Middle cerebral artery
EB: Evans blue
