# Supplementary material for: Apelin-13/APJ system attenuates early brain injury via suppression of endoplasmic reticulum stress-associated TXNIP/NLRP3 inflammasome activation and oxidative stress in a AMPK-dependent manner after subarachnoid hemorrhage in rats
Source: J Neuroinflammation. 2019 Dec 2;16:247. doi: 10.1186/s12974-019-1620-3 (PMC6889224; doi:10.1186/s12974-019-1620-3)
Supplement: Supplementary file 1 — Additional file 1: Table S1 Study design and animal usage. Table S2. Rats physical data after surgeries. Figure S1. The ventral side was divided into six parts. SAH severity score: a grade from 0-3 is dependent on the amount of blood clot in each segment as follows: grade 0:no blood clot; 1: minimal blood clot; 2: moderate blood clot with recognizable arteries; 3: blood clot obliterating all arteries. The SAH grade was the total scores of the six parts, with minimal score of 0 and maximal score of 18. Figure S2. Effects of apelin-13 on brain edema: the quantification of brain water content of cerebellum and brain stem at 24 h after SAH. Figure S3. Depletion Efficiency of APJ siRNA with Naïve Rats. (A) Representative Western blot images. (B) Quantitative analyses of APJ. n=6 for each group. The bars represent the mean ± SD. *p<0.05 versus naïve. [file 12974_2019_1620_MOESM1_ESM.docx]

**Table S1** Study design and animal usage.

| Part | Groups | long-term | Brain Edema | EB | WB | ROS | IF | mortality(SAH) | excluded(SAH) | Sum |
| --- | --- | --- | --- | --- | --- | --- | --- | --- | --- | --- |
| Exp. 1 | Sham | - | - | - | 6 | - | 2 | 39/241(16.18%) | 9 | 312 |
|  | SAH (3h, 6h, 12h, 24h, 48h, 72 h) | - | - | - | 36 | - | 2(24h) |  |  |  |
| Exp. 2 | Sham | - | 6 | 6 | - | - | - |  |  |  |
|  | SAH + vehicle | - | 6 | 6 | - | - | - |  |  |  |
|  | SAH + apelin-13 (15μg/kg) | - | 6 | 6 | - | - | - |  |  |  |
|  | SAH + apelin-13 (50μg/kg) | - | 6 | 6 | - | - | - |  |  |  |
|  | SAH + apelin-13 (150μg/kg) | - | 6 | 6 | - | - | - |  |  |  |
| Exp. 3 | Sham | 10 | - | - | - | - | - |  |  |  |
|  | SAH + vehicle | 10 | - | - | - | - | - |  |  |  |
|  | SAH + apelin-13 (50μg/kg) | 10 | - | - | - | - | - |  |  |  |
| Exp. 4 | Sham | - | - | - | 6 | 6 | - |  |  |  |
|  | SAH + vehicle | - | - | - | 6 | 6 | - |  |  |  |
|  | SAH + apelin-13 (50μg/kg) | - | - | - | 6 | 6 | - |  |  |  |
|  | SAH + apelin-13 + scramble siRNA | - | - | - | 6 | 6 | - |  |  |  |
|  | SAH + apelin-13 +APJ siRNA | - | - | - | 6 | 6 | - |  |  |  |
| Exp. 5 | Sham | - | - | - | 6 | - | 5 |  |  |  |
|  | SAH + vehicle | - | - | - | 6 | - | 5 |  |  |  |
|  | SAH + apelin-13 (50μg/kg) | - | - | - | 6 | - | 5 |  |  |  |
|  | SAH+ apelin-13 + dorsomorphin | - | - | - | 6 | 6 | 5 |  |  |  |
| Exp. 6 | naïve | - | - | - | 6 | - | - |  |  |  |
|  | naïve + scrRNA | - | - | - | 6 | - | - |  |  |  |
|  | naive + APJ siRNA | - | - | - | 6 | - | - |  |  |  |
|  | Subtotal | 30 | 30 | 30 | 114 | 36 | 24 | 39 | 9 |  |

SAH, subarachnoid hemorrhage; EB, evans blue; WB, western blotting; IF, immunofluorescence.

| **Table S2**. Rats physical data after surgeries. | | | | | | | |
| --- | --- | --- | --- | --- | --- | --- | --- |
| Group | Rats used | T(℃) | HR(/min) | BP(mmHg) | BG(mmol/L) | PO2(mmHg) | PCO2(mmHg) |
| Sham | 53 | 37.4±0.18 | 361±7.9 | 132±0.25 | 6.6±0.32 | 76±3.8 | 38±2.2 |
| SAH | 38 | 37.6±0.21 | 359±8.8 | 133±0.89 | 6.5±0.49 | 73±2,5 | 40±1.9 |
| SAH+vehicle | 45 | 38.0±0.21 | 361±7.8 | 127±0.93 | 6.5±0.42 | 72±3.8 | 38±2.5 |
| SAH+apelin-13 | 69 | 37.8±0.18 | 362±8.0 | 135±1.3 | 6.4±0.47 | 73±2.9 | 39±2.7 |
| SAH+apelin-13+scramble siRNA | 12 | 37.8±0.25 | 351±8.1 | 130±0.90 | 6.6±0.52 | 74±5.0 | 42±2.1 |
| SAH+apelin-13+dorsomorphin | 17 | 37.6±0.16 | 360±9.0 | 133±0.87 | 6.7±0.36 | 74±4.3 | 40±2.6 |
| SAH+apelin-13+APJ siRNA | 12 | 37.5±0.21 | 352±5.4 | 128±0.82 | 6.5±0.34 | 75±5.1 | 39±2.5 |

T, temperature; HR, heart rate; BP, blood pressure; BG, blood glucose.

**
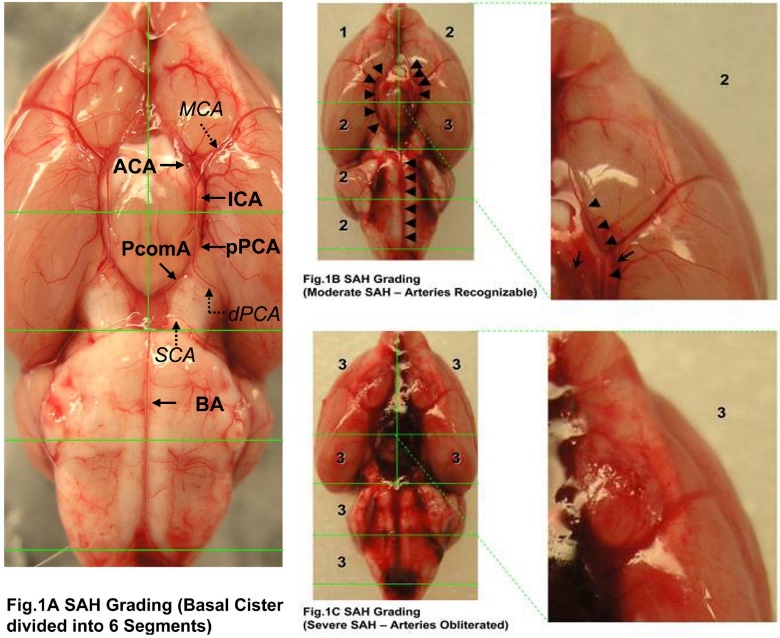
**

**（Sugawara T, et al. J Neurosci Methods. 2009.[1]）**

**Figure S1** The ventral side was divided into six parts. SAH severity score: a grade from 0-3 is dependent on the amount of blood clot in each segment as follows: grade 0:no blood clot; 1: minimal blood clot; 2: moderate blood clot with recognizable arteries; 3: blood clot obliterating all arteries. The SAH grade was the total scores of the six parts, with minimal score of 0 and maximal score of 18.


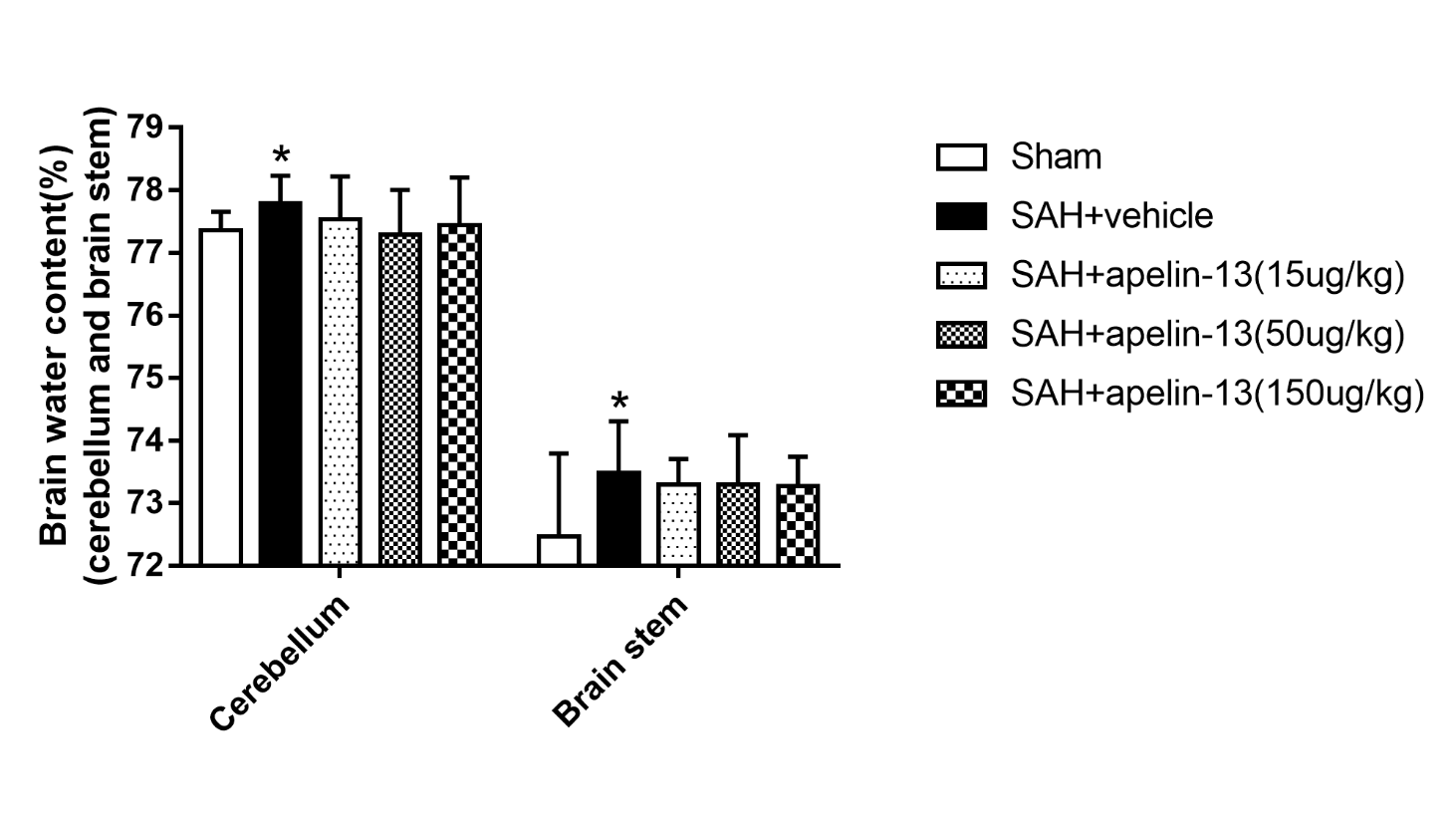


**Figure S2.** Effects of apelin-13 on brain edema: the quantification of brain water content of cerebellum and brain stem at 24 h after SAH.


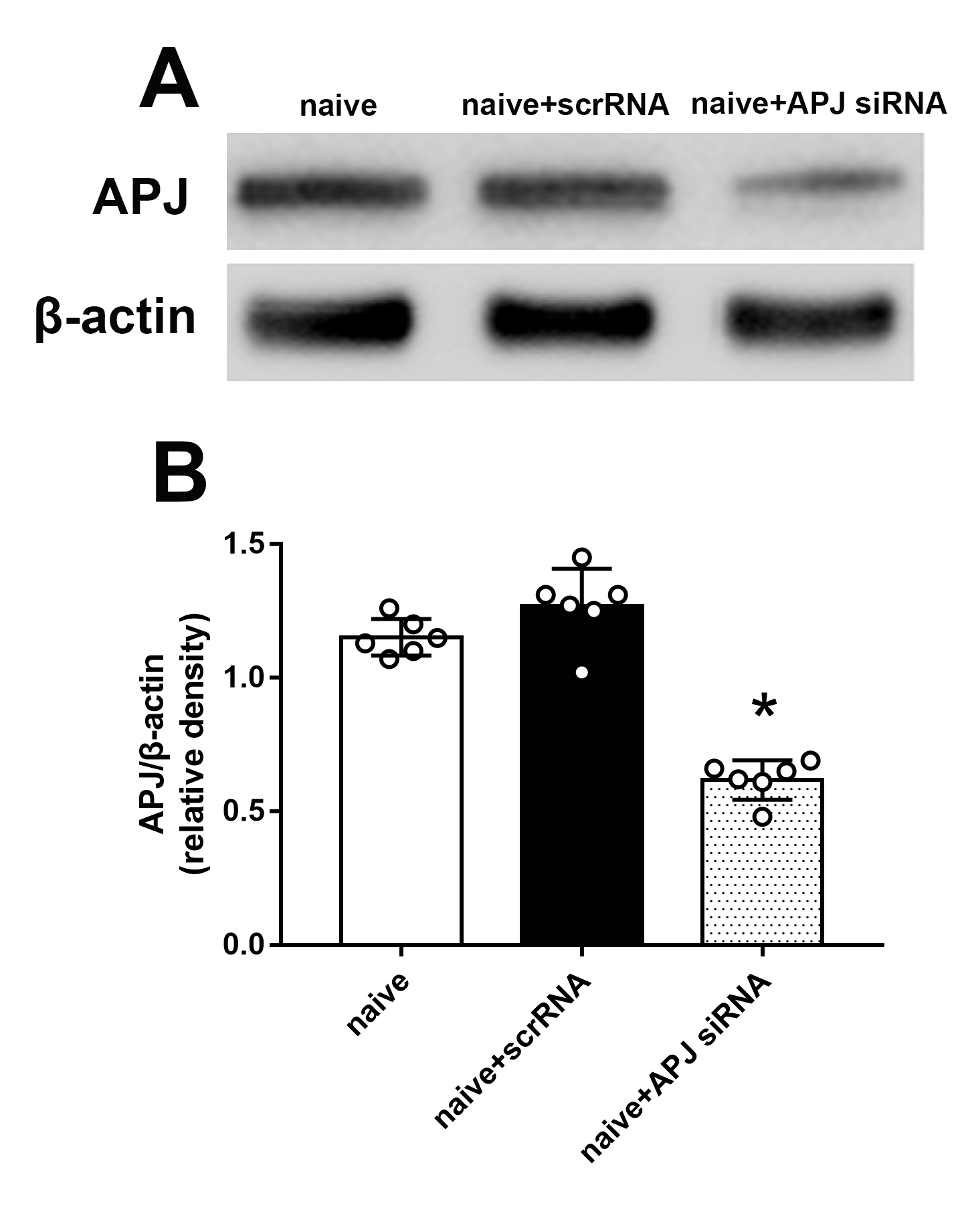


**Figure S3** Depletion Efficiency of APJ siRNA with Naïve Rats. (A) Representative Western blot images. (B) Quantitative analyses of APJ. n=6 for each group. The bars represent the mean ± SD. *p<0.05 versus naïve.

1. Sugawara, T., et al. A new grading system evaluating bleeding scale in filament perforation subarachnoid hemorrhage rat model. J Neurosci Methods. 2008; 167(2): 327-34.
